# Leadership Development for Hospital Physicians

**DOI:** 10.5334/jbr-btr.1416

**Published:** 2017-12-16

**Authors:** Bart Claikens

**Affiliations:** 1 AZ Damiaan Oostende, BE

Physicians are highly specialized professionals educated in medicine and key actors in a hospital organization. High-quality care and patient safety is their core business and main objective.

However, working in complex healthcare organizations requires significantly more than just specialized medical skills.

The rapidly changing field of the current healthcare industry and the complexity of hospitals demand that physicians also engage in leadership and management.

The hospital environment is evolving according to the shifting needs of patients. An aging population, chronic pathology, and multi-morbidity results in an evolution from cure- to care-based and patient-centralized healthcare.

Moreover, the healthcare environment currently faces significant financial hurdles. Long-term care has risen over the past few decades and is expected to increase in the coming years. New medical technologies improve diagnosis and treatment, but they also increase spending in healthcare. Wise budget use in combination with operational performance and creating a culture of value-based decision-making is undeniably a key goal for current leading physicians.

Hospital and physician alignment is more crucial than ever, given the shrinking budgets and call for transparency and increased efficiency. Both parties need a deeper integration that moves beyond the current state of affairs.

This evolution increases complexity on several fronts and calls for an open mindset, which means that operational excellence, lean management, and business intelligence must form the solid foundation for ongoing improvements in clinical and service quality. Several strategic challenges also dot the landscape of hospital caregivers targeting mergers and consolidation of hospital geographical networks.

Hospitals need talented, educated and engaged physicians as leaders and managers. Good and flexible leadership behavior will result in better quality of care. These leading physicians must be able to cope with the fundamental challenges facing Belgian hospitals, to include nurturing a culture that embraces their vision and mission, and provision of accessible, affordable, high-quality and safe patient care.

The leadership skills of physicians with a supervisory/authoritative role are reflected at both organizational and personal levels in the optimization of human and organizational capital. Their leadership style is enhanced through regular interaction with aides, peers, corporations, board members, and other stakeholders and is appreciated because of shared strategy and an integral mission.

On the macro- and micro-level of hospital organizations, the senior hospital management, its physicians and other caregivers must cope with looming changes to their environment, targeted at improving the quality and efficiency of care in hospitals. In the macro-environment, hospitals must remain conscious of the financial implications for their operations, and possess strategic insight into pending hospital consolidation and networking. Their value-based management implies a common morality in senior management and physician alignment. In the micro-environment, the hospital system is rapidly evolving with growing medical teams, the pursuit of hospital accreditation and shifting patient needs.

In this paper, we critically reflect on the background of and environmental prospects for the Belgian healthcare system, including its mission, performance indicators and spending. The constraints and the opportunities of Belgian healthcare are investigated and current and future challenges are addressed. In this setting, we will investigate how sufficient physician leadership is essential to reacting and adapting to upcoming healthcare changes and for the provision of quality care.

A review of the international literature on leadership styles took place using online search engines, academic management journals, reference books and business school outlines in order to answer the question: what should leading physicians do?

Personal and interactive interviews with hospital senior managers in Flanders are addressed to investigate the need for physicians with leadership skills and for alignment with hospital stakeholders.

A dedicated online survey was conducted to address leading physicians in Flander’s general and university hospitals. This survey of leading physicians provided a clear overview of their profiles, behavior, educational background and development needs.

A survey of senior managers in for-profit companies provided relevant information on their leadership styles, experiences and managerial education.

We will investigate the current leading physicians’ characteristics and their managerial knowledge and education in order to see where and how added value can be provided in their overall leadership development.

On the one hand, involving the hospital’s own leading physicians in strategic planning, policy making, and other governance related activities seems to be the key to a successful realization of its mission. On the other, however, we will demonstrate that there is a demand for trained, educated and experienced physicians in leadership and management, which will only intensify in the future.

Based on the shortage of physicians with managerial knowledge and skills, and the lack of dedicated managerial education in medical school and during their professional careers as physicians, the rationale behind demand for leadership and management support and education is clear.

As a result, we propose a dedicated, customized leadership and management development program for physicians to be planned and implemented in partnership with business schools. Moreover, we suggest that a medical management educational platform and organization be set up in support of senior hospital managers and leading physicians.

This paper hopes to engage physicians in leadership and encourage them toward ongoing managerial education and attendance of leadership development programs.

